# *Boehmeria nivea* Attenuates the Development of Dextran Sulfate Sodium-Induced Experimental Colitis

**DOI:** 10.1155/2014/231942

**Published:** 2014-06-17

**Authors:** Eun Ju Shin, Mi Jeong Sung, Hye Jeong Yang, Myung Sunny Kim, Jin-Taek Hwang

**Affiliations:** ^1^Department of Food Biotechnology, University of Science & Technology, 217 Gajeong-ro, Yuseong-gu, Daejeon 305-333, Republic of Korea; ^2^Korea Food Research Institute, 1201 Anyangpangyoro, Bundang-gu, Seongnam-si, Gyeonggi-do 463-746, Republic of Korea

## Abstract

We examined the therapeutic effect of an ethanol extract derived from *Boehmeria nivea* (Linn.) Gaudich in a mouse model of experimental colitis. Treatment with 70% ethanol extract derived from *B. nivea* (EBN) at a dose of 100, 200, or 500 mg/(kg*·*d) improved colon shortening, body weight, the disease activity index (DAI), and histopathological score of DSS-induced colitis mice. DSS significantly increased the levels of cyclooxygenase-(COX-) 2 in colon tissue relative to that of the untreated control group. EBN administered at 100, 200, or 500 mg/(kg*·*d) reduced COX-2 levels in the DSS-treated mice. In addition, EBN decreased the DSS-induced secretion of the inflammatory cytokine interleukin-6 (IL-6) and chemokine monocyte chemotactic protein-1 (MCP-1). Taken together, these data suggest that *B. nivea* extract is effective in preventing colitis.

## 1. Introduction 

Inflammatory bowel diseases (IBD), such as ulcerative colitis (UC), are inflammatory diseases. Several anti-inflammatory drugs including sulfasalazine, steroids, and nonsteroidal anti-inflammatory drugs have been used to treat UC [1]; however, these drugs have limited effectiveness, because of their associated side effects and the varying etiology of UC. Experimental colitis models have been used to identify therapeutic agents and elucidate the underlying physiologic mechanisms of UC [2]. The widely employed DSS-induced colitis model recapitulates the histological characteristics of UC [2]. Administration of DSS induces colitis in the rectum that spreads to the anus, resulting in inflammatory changes including crypt distortion, ulceration, and the infiltration of inflammatory cells [2].

Numerous investigators, who have demonstrated that immune dysfunction contributes to the development of UC, have emphasized the importance of COX in UC pathogenesis. COX has three isoforms, COX-1, -2, and -3 [3]. In mammalian cells, COX-1 is constitutively expressed; however, COX-2 expression is induced by the inflammatory response. Recently, COX-3 was shown to be encoded by the same gene as COX-1. The inflammatory response is stimulated by pathogenic infections, resulting in the production of proinflammatory cytokines and chemokines [4]. In IBD patients, proinflammatory cytokines and chemokines such as IL-6 and MCP-1 are upregulated in intestinal mucosa [4]. Thus, inhibiting cytokine production through the use of therapeutic agents is a potential strategy for alleviating the symptoms of IBD.

Natural agents have been proposed by several investigators as a treatment of many diseases including IBD. Mounting evidence shows that the therapeutic efficacy of these natural agents relies on their ability to reduce cytokine production and COX-2 levels in vitro and in vivo [5]. For example,* Serpylli herba* extract reduces both trinitrobenzene sulfonic acid- (TNBS-) or DSS-induced colitis by reducing the levels of tumor necrosis factor- (TNF-) *α* and IL-6 cytokines and the chemokine MCP-1 [5].* Solanum tuberosum* L. cv Jayoung epidermis, a color-fleshed potato, reportedly inhibits the expression of COX-2 and reduces the severity of colitis in DSS-induced mice [6].

*Boehmeria nivea* (Linn.) Gaudich (Urticaceae) is a flowering plant that is traditionally used to treat several diseases and heal wounds in Asian countries including China, Korea, the Philippines, and Thailand [7, 8]. Previously, we showed that* B. nivea* reduces lipopolysaccharide- (LPS-) induced secretion of proinflammatory cytokines such as TNF-*α* and IL-6 through the inhibition of p38 and c-Jun N-terminal kinase (JNK) [8]; however, the ability of* B. nivea* to control the effects of DSS-induced colitis has not been investigated. The results of our previous work prompted us to determine whether* B. nivea* exhibits an anti-inflammatory effect in DSS-induced colitis model mice.

## 2. Materials and Methods

### 2.1. Reagents

The COX-2 and *β*-actin antibodies were obtained from Cayman Chemical (Ann Arbor, MI, USA) and Cell Signaling Technology (Beverly, MA, USA), respectively. Horseradish peroxidase-conjugated secondary antibody was obtained from Santa Cruz Biotechnology (Santa Cruz, CA, USA). DSS was purchased from MP Biochemicals (Aurora, OH, USA).

### 2.2. Preparation of* B. nivea* Extract

*B. nivea* leaves were provided by the Seocheon County Office (Seocheon, Republic of Korea), where a voucher specimen had been deposited. EBN was prepared from leaves as described previously [8].

### 2.3. Experimental Colitis

Experimental colitis was induced by 3% DSS as described previously [9]. Briefly, 8-week-old male C57BL/6 mice were obtained from Charles River Korea (Seoul, Republic of Korea) and were housed at a temperature of 22–26°C under a 12 h light/dark cycle with free access to water. After a 1-week period of adaptation, the mice were randomly divided into the following 5 groups (8 mice/group): untreated (no DSS), DSS-treated (3% DSS), DSS + EBN 100 (3% DSS and EBN at 100 mg/kg body weight per day), DSS + EBN 200 (3% DSS and EBN at 200 mg/(kg*·*d)), and DSS + EBN 500 (3% DSS and EBN at 500 mg/(kg*·*d)). The untreated group received tap water without DSS for 8 days. DSS alone and DSS + EBN were orally administered for 7 days.

The mice were scored daily with respect to body weight, stool formation, and fecal occult blood. The weight loss, stool formation, and fecal occult blood scores were averaged to determine the DAI. Scores were assigned as follows: weight change (0: <1%, 1: 1–5%, 2: 5–10%, 3: 10–15%, or 4: >15%), fecal occult blood (0: negative, 2: positive, or 4: gross bleeding), and stool formation (0: normal, 2: loose stool, or 4: diarrhea). Scoring was performed as previously described [9]. On day 7, the mice were sacrificed by cardiac puncture under ketamine/xylazine anesthesia and their colon tissues were isolated.

### 2.4. MCP-1 and IL-6 Quantification

Blood samples were collected and IL-6 and MCP-1 levels were measured using enzyme-linked immunosorbent assay (ELISA) kits (R&D Systems, Minneapolis, MN, USA) according to the manufacturer's instructions.

### 2.5. Histopathological Analysis

Paraffin-embedded colon sections, fixed in formalin, were stained using hematoxylin and eosin (H&E). The degree of colitis was assessed with respect to the presence of edema, extent of injury, leukocyte infiltration, crypt damage, and loss of goblet cells as described previously [9]. These criteria were scored as follows: inflammation severity (0: none, 1: slight, 2: moderate, or 3: severe), extent of injury (0: none, 1: mucosal, 2: mucosal and submucosal, or 3: transmural), and crypt damage (0: none, 1: damage to the basal third of the crypt, 2: damage to the basal two-thirds of the crypt, 3: only surface epithelium intact, or 4: loss of entire crypt and epithelium).

### 2.6. Protein Extraction and Western Blot Analysis

Cells were lysed with lysis buffer (50 mM Tris-HCl, pH 8.0, 5 mM EDTA, 150 mM NaCl, 1% NP-40, 1 mM PMSF, protease inhibitor cocktail, and phosphatase inhibitor cocktail). Total protein was collected and then separated using SDS-polyacrylamide gel electrophoresis. The protein was transferred to the nitrocellulose paper and detected by western blot analysis using specific antibodies.

### 2.7. Statistical Analysis

Statistical differences of the results were analyzed by Student's *t*-test between groups. Data show the mean ± standard deviation (SD) of at least 3 independent experiments performed in triplicate. *P* < 0.05 was considered significant.

## 3. Results

### 3.1. EBN Improves Colon Shortening, DAI, and Body Weight in DSS-Induced Colitis Mice

**Figure 1:**
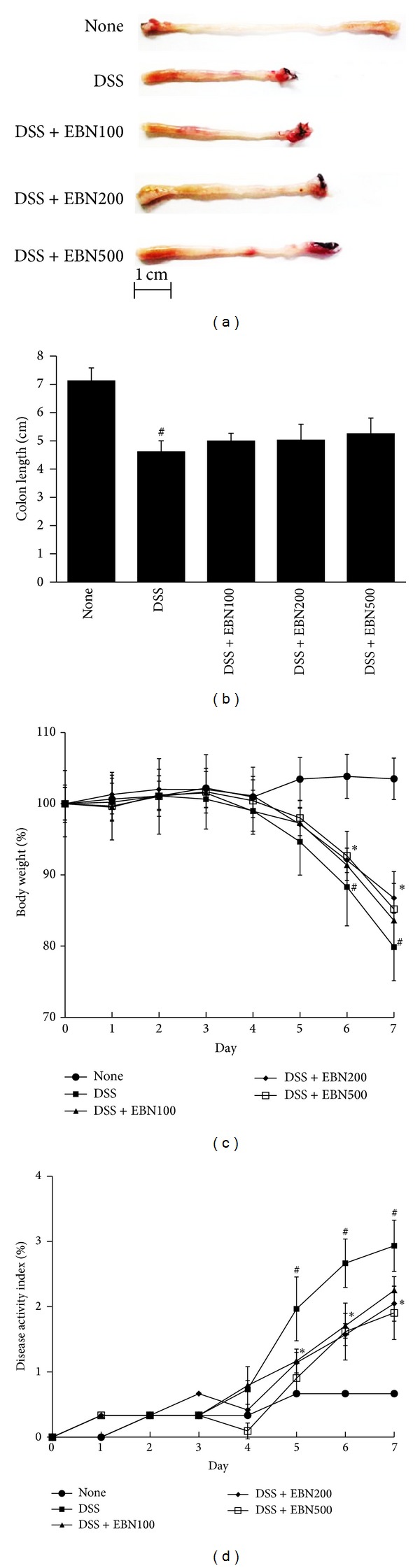
The effect of ethanol extract derived from* B. nivea* (EBN) on colon length, body weight, and disease activity index (DAI) in dextran sulfate sodium- (DSS-) induced colitis mice. Mice colons were isolated 7 days after DSS administration and their lengths were measured ((a) and (b)). Body weight was measured daily (c). Clinical scores of each mouse were monitored daily (d). Data from 3 independent experiments is shown and expressed as the mean ± SD (*n* = 8 per group). *P < 0.05 versus DSS-treated; ^#^ *P* < 0.05 versus untreated.

Our previous work suggested that* B. nivea* has an anti-inflammatory effect on LPS-induced RAW264.7 macrophages by reducing cytokine levels [8]. Thus, we investigated whether* B. nivea* has an inhibitory effect on an IBD, UC, by using an experimental colitis mouse model. As shown in Figures 1(a) and 1(b), treatment with 3% DSS reduced the colon length, body weight, and DAI relative to that of the untreated group. Administration of EBN mildly reduced colon shortening in the DSS-treated group. The reduction in body weight caused by the 3% DSS treatment was significantly improved by administering EBN at 100, 200, and 500 mg/(kg*·*d) (Figure 1(c)). The 3% DSS treatment caused diarrhea and rectal bleeding, two criteria that were scored and included in the DAI (Figure 1(d)). The DAI was reduced in mice that had been administered EBN at 100, 200, and 500 mg/(kg*·*d).

### 3.2. Histopathological Scores of DSS-Induced Colitis Mice Are Reduced by Administering EBN

**Figure 2:**
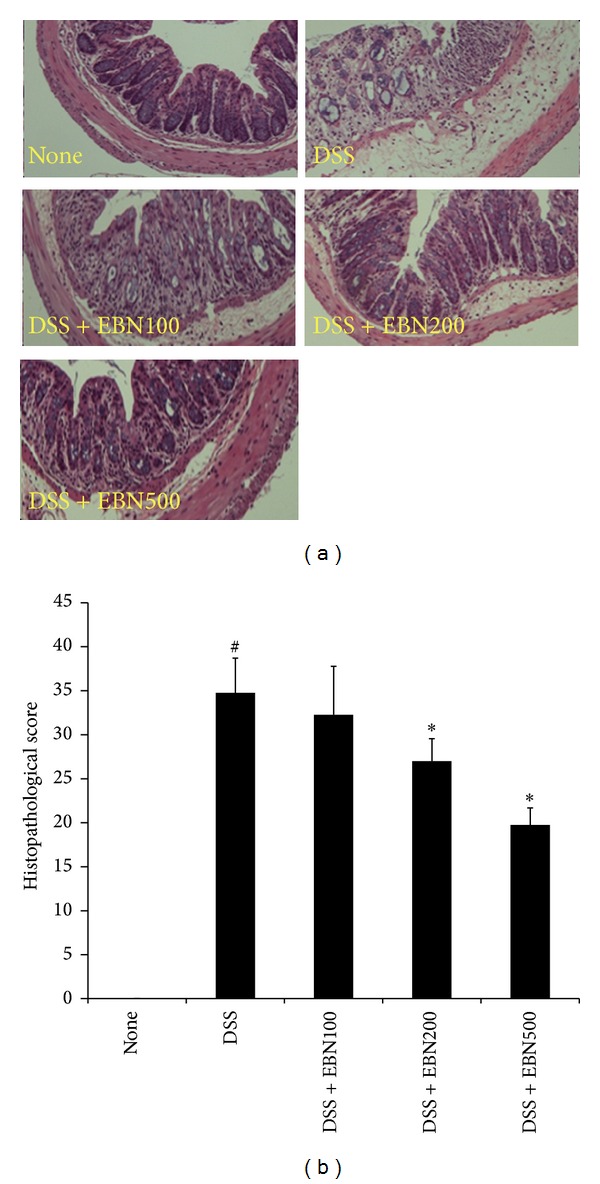
The effect of EBN on the histopathological score in DSS-induced colitis mice. Mice colons were obtained at 7 days post-DSS administration and were stained with H&E. H&E staining was performed on colon sections obtained from mice belonging to the untreated (no DSS), DSS-treated (3% DSS), DSS + EBN 100 (3% DSS and EBN at 100 mg/kg body weight per day), DSS + EBN 200 (3% DSS and EBN at 200 mg/kg*·*d), and DSS + EBN 500 (3% DSS and EBN at 500 mg/kg*·*d) groups. Images were obtained at 40x magnification (a). Histopathological scores were analyzed from slides (b). Data from 3 independent experiments is shown and expressed as the mean ± SD (*n* = 8 per group). *P < 0.05 versus DSS-treated; ^#^ *P* < 0.05 versus untreated.

We next performed a histological analysis by performing H&E staining. As shown in Figures 2(a) and 2(b), 3% DSS treatment induced an inflammatory response that affected colonic architecture, cell infiltration, crypt shortening or loss, and goblet cell depletion. Administration of EBN significantly reduced the number of infiltrating cells, mucosal injury, and edema. We scored the severity of the inflammation (see Materials and Methods). Mice treated with 3% DSS showed higher histopathological scores than those belonging to the untreated group. The histopathological scores were significantly improved when EBN was administered at 100, 200, and 500 mg/(kg*·*d) (Figure 2(b)).

### 3.3. EBN Decreases the Levels of IL-6, MCP-1, and COX-2 in DSS-Induced Colitis Mice

**Figure 3:**
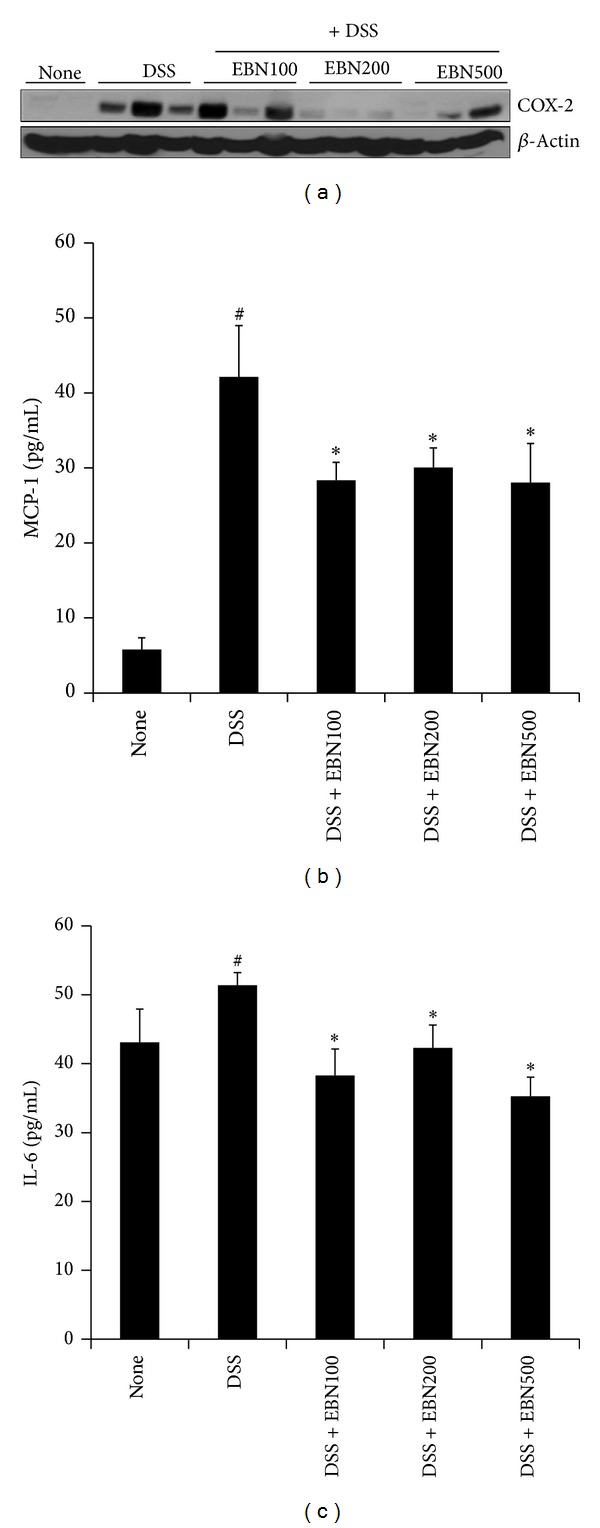
The effect of EBN on IL-6, MCP-1, and COX-2 levels in DSS-induced colitis mice. COX-2 levels were measured in colon tissue lysates by using western blot analysis (a). *β*-Actin was used as an internal control. Serum levels of IL-6 and MCP-1 were measured using enzyme-linked immunosorbent assays (ELISAs) ((b) and (c)). The levels of IL-6, MCP-1, and COX-2 are shown for the untreated, DSS-treated, DSS + EBN 100, DSS + EBN 200, and DSS + EBN 500 groups. All data are expressed as the mean ± SD. *P < 0.05 versus DSS-treated; ^#^ *P* < 0.05 versus untreated.

The inflammatory cytokine IL-6 and chemokine MCP-1 play central roles in inflammation [5]. Furthermore, the development of inflammatory diseases such as UC is mediated by COX-2. Thus, we examined whether EBN reduces IL-6, MCP-1, and COX-2 levels in DSS-induced colitis mice. As shown in Figure 3(a), DSS treatment significantly increased the levels of COX-2 in colon tissue relative to that of the untreated control group. Administration of EBN (200 mg/kg and 500 mg/kg) reduced the COX-2 levels that had been elevated in response to DSS treatment. DSS treatment also significantly increased the production of serum IL-6 and MCP-1 relative to controls (Figures 3(b) and 3(c)). Administration of EBN significantly decreased the production of IL-6 and MCP-1 in DSS-treated group (Figures 3(b) and 3(c)). The maximal effect of EBN was obtained with 100 mg/kg. These results suggest that EBN has an anti-inflammatory effect in mice with induced UC, which is mediated, at least in part, by reducing the levels of COX-2, IL-6, and MCP-1.

## 4. Discussion

Previously, we showed that ethanol extract of* B. nivea* exerts an anti-inflammatory effect through inhibiting p38 and JNK in LPS-induced RAW264.7 macrophage cells [8]. As an extension of that study, we examined the anti-inflammatory efficacy of* B. nivea* in an experimental colitis mouse model. We found that* B. nivea* improves DSS-induced experimental colitis. EBN was able to reduce colon shortening, weight loss, DAI, and histopathological scores associated with DSS treatment (Figures 1 and 2). Furthermore, COX-2 levels were reduced by administering EBN to DSS-treated mice (Figure 3).

*B. nivea* has traditionally been used in Asian countries to prevent diseases and reduce inflammation [7, 8]. Numerous studies have reported that* B. nivea* is a bioactive agent, exhibiting antifungal and antioxidant properties [7, 8, 10]. The functional properties of natural agents are thought to be associated with their polyphenol content [11]. Our previous work suggests that plant polyphenols possess beneficial effects on health [12].* B. nivea* contains several polyphenol compounds including chlorogenic acid, rutin, luteolin-7-glucoside, naringin, hesperidin, and tangeretin [8]. Thus, we speculated that* B. nivea* was able to reduce the inflammatory response and that polyphenol compounds are responsible for this effect. Several medications, including sulfasalazine, have been used to treat patients with IBD including UC [1]. Steroids or nonsteroidal anti-inflammatory drugs have also been used to treat IBD [1]. However, these drugs can cause severe side effects in patients. Thus, investigators have proposed that alternative medicines and natural products that exhibit similar activity to sulfasalazine can be used to treat IBD [13, 14]. For example, plant-derived polyphenols have been reported to improve UC. A bioflavonoid derived from the seeds of* Garcinia kola*, kolaviron, exhibits an anticolitis effect by reducing nitric oxide, myeloperoxidase activity, TNF-*α*, and IL-1*β* in DSS-induced colitis mice [13]. These effects of kolaviron were comparable to that of sulfasalazine. The flavonoid hesperidin has also been shown to reduce DAI and IL-6 levels in DSS-induced colitis mice [14]. Sulfasalazine reduced DAI and IL-6 level in DSS-induced colitis mice under the same conditions, suggesting that hesperidin can be used to prevent colitis. Furthermore, cytokines including IL-6 were a critical target of both hesperidin and sulfasalazine treatments [14]. Leucocyte infiltration induces mucosal tissue damage in colons, resulting in an inflammatory response involving cytokines and the chemokine MCP-1 [15]. Anticolitis medications such as sulfasalazine significantly decrease colitis and neutrophil/macrophage infiltration by reducing cytokine and chemokine levels [16, 17]. Polyphenol-rich extra-virgin olive oil has been shown to modulate macrophage activation and decrease cytokine production in UC [18]. In this study, DSS increased the serum levels of IL-6 and MCP-1 relative to control mice (Figures 3(b) and 3(c)). Interestingly, administration of EBN significantly decreased the production of both IL-6 and MCP-1 in the DSS-treated group (Figures 3(b) and 3(c)). These data are consistent with the previous work that suggests sulfasalazine-like natural agents act as inhibitors of cytokine production in UC. Therefore, we hypothesize that EBN is a suppressor of cytokine production and blocks leucocyte infiltration in UC, in a manner similar to sulfasalazine. However, we were unable to elucidate the precise mechanism by which EBN inhibits cytokine production in DSS-induced colitis mice. We also found that administration of EBN reduced the levels of COX-2 that had been increased by DSS treatment (Figure 3(a)). COX-2 is a considerable target for the UC that treatment of DSS induces strong COX-2 expression in colon tissue [19]. Selective COX-2 inhibitors exert a therapeutic effect towards both TNBS- and DSS-induced colitis by reducing cytokine levels [20]. In this study, we demonstrated that DSS treatment significantly increased the levels of COX-2 in colon tissue (Figure 3(a)), suggesting that COX-2 levels are upregulated in the DSS-induced colitis model. Several studies report that polyphenol-rich extra-virgin olive oil downregulates COX-2 levels [18] and the polyphenol resveratrol decreases COX-2 levels in experimental colitis models [21], suggesting that polyphenols possess anticolitis activity. Here, we demonstrate that EBN, rich in polyphenols such as chlorogenic acid, rutin, luteolin-7-glucoside, naringin, hesperidin, and tangeretin, reduces the levels of COX-2 that were elevated as a result of DSS treatment (Figure 3(a)). Therefore, we hypothesize that polyphenols may be a potential factor for the reduction of colitis by EBN.

## 5. Conclusion

We demonstrated that an ethanol extract of* B. nivea* exerts an inhibitory effect on colitis pathogenesis, improving colon shortening, body weight, DAI, and histopathological scores in DSS-induced colitis mice. Furthermore, we found that COX-2 levels were reduced by EBN in colon tissues. These findings suggest that* B. nivea* is effective in preventing IBD including UC. Our findings contribute to the understanding of the potential functionality and underlying mechanisms of* B. nivea* bioactivity.
